# Treatment strategies for reflux esophagitis including a potassium‐competitive acid blocker: A cost‐effectiveness analysis in Japan

**DOI:** 10.1002/jgf2.429

**Published:** 2021-02-21

**Authors:** Yasuki Habu, Ryuhei Hamasaki, Motonobu Maruo, Tatsuya Nakagawa, Yuki Aono, Daisaku Hachimine

**Affiliations:** ^1^ Department of Gastroenterology Saiseikai‐Noe Hospital Osaka Japan

**Keywords:** cost‐effectiveness, gastroesophageal reflux disease, potassium‐competitive acid blocker, proton pump inhibitor, reflux esophagitis, vonoprazan

## Abstract

**Introduction:**

Gastroesophageal reflux disease is a common condition, and proton pump inhibitors (PPIs) are the mainstays of treatment. However, concerns have been raised about the safety of PPIs. A potassium‐competitive acid blocker (P‐CAB), vonoprazan (VPZ), was recently introduced, which may provide clinical benefits. This study was performed to investigate the cost‐effectiveness of alternative long‐term strategies including continuous and discontinuous treatment with VPZ for the management of reflux esophagitis in Japan.

**Methods:**

A health state transition model was developed to capture the long‐term management of reflux esophagitis. Four different strategies were compared: (a) intermittent PPI using lansoprazole (LPZ); (b) intermittent P‐CAB; (c) maintenance PPI using LPZ; and (d) maintenance P‐CAB.

**Results:**

Intermittent P‐CAB was the most cost‐effective, and the number of days for which medication was required with this strategy was fewest. Maintenance PPI was more efficacious, but more costly than intermittent P‐CAB. Maintenance P‐CAB was more efficacious, but more costly than maintenance PPI. Co‐payments were higher for maintenance PPI than for intermittent P‐CAB, and for maintenance P‐CAB than for maintenance PPI, which were considered reasonable for the majority of patients to improve symptoms.

**Conclusions:**

Intermittent P‐CAB appears to be the strategy of choice for the majority of reflux esophagitis patients in clinical practice. If a patient is not satisfied with the symptom control of the current strategy, switching to a more effective strategy appears to be a reasonable option for the majority of patients.

## Graphical abstract

A decision analysis was conducted to investigate the cost‐effectiveness of alternative long‐term strategies for the management of reflux esophagitis in Japan. Intermittent treatment with a potassium‐competitive acid blocker, vonoprazan, appears to be the strategy of choice for the majority of reflux esophagitis patients in clinical practice. If a patient is not satisfied with the symptom control of the current strategy, switching to a more effective strategy appears to be a reasonable option for the majority of patients.

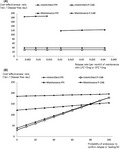

## 1 INTRODUCTION

Gastroesophageal reflux disease (GERD) is a common condition presenting to primary care physicians, which diminishes a patient's quality of life and reduces work productivity.^1^, ^2^, ^3^, ^4^ GERD is the most common gastrointestinal‐related diagnosis, and the expenses associated with its treatment have a significant impact upon healthcare costs.^1^

Proton pump inhibitors (PPIs) are efficacious for symptom resolution and mucosal healing of erosive esophagitis, and they are cost‐effective.^3^, ^5^, ^6^ Thus, the administration of a standard dose of PPIs for 8 weeks is recommended as an initial treatment for GERD.^3^, ^7^ PPI maintenance therapy is also efficacious, and recommended as an option for the long‐term management.^3^, ^7^ However, many patients with GERD do not take PPIs on a daily basis even when they are prescribed as such.^8^, ^9^ Clinical studies suggested that a proportion of patients may be managed effectively by discontinuous treatments such as intermittent or on‐demand therapies.^10^, ^11^, ^12^ Recent studies have linked PPI use to serious adverse effects, and safety issues associated with PPI have attracted widespread attention.^13^ Although it is uncertain whether associations between PPI use and potential side effects are causal, the potential has forced physicians to carefully consider the safety of long‐term PPI use. This topic was included in the American Board of Internal Medicine Foundation's Choosing Wisely campaign.^14^

The cost of ongoing medical care for GERD is substantial.^1^ Therefore, treatment strategies for GERD should be cost‐effective. Moreover, in daily clinical practice, the management of GERD should consider the patients' perspective or preferences, involving, for example, the inconvenience of taking medication, the fear of side effects from long‐term therapy, or willingness to pay out‐of‐pocket expenses for access to a more effective but more expensive medication.^15^ Indeed, PPI maintenance therapy is recommended as an option for the long‐term management of GERD,^3^, ^7^ but in reality, patients prescribed long‐term medications for GERD often take them only when symptoms recur.^8^, ^9^

A potassium‐competitive acid blocker (P‐CAB), vonoprazan (VPZ), was approved for the treatment of reflux esophagitis in 2015 in Japan. VPZ has been reported to achieve a more rapid and profound suppression of gastric acid secretion than PPIs.^16^ Clinical studies demonstrated the remarkably high efficacy of VPZ when used as either a healing or maintenance therapy.^17^, ^18^, ^19^ A recent cost‐effectiveness analysis revealed that VPZ is more effective and less costly than lansoprazole (LPZ), a PPI for the acute‐phase treatment of reflux esophagitis.^20^

This study describes a clinical decision analysis to compare the cost‐effectiveness of alternative long‐term strategies for the management of erosive esophagitis in the era of a novel P‐CAB (VPZ) in Japan. The overall healthcare budget is chosen as the primary perspective. The self‐pay burden of patients is also considered. Several clinical outcomes are described.

## 2 MATERIALS AND METHODS

### 2.1 Clinical starting points and strategies

The principal decision is to treat patients with healed erosive reflux esophagitis, who are in remission, with alternative management strategies. Four different strategies were compared: (a) intermittent treatment with LPZ; (b) intermittent treatment with VPZ; (c) maintenance treatment with LPZ; and (d) maintenance treatment with VPZ.

### 2.2 Analytic model

**FIGURE 1:**
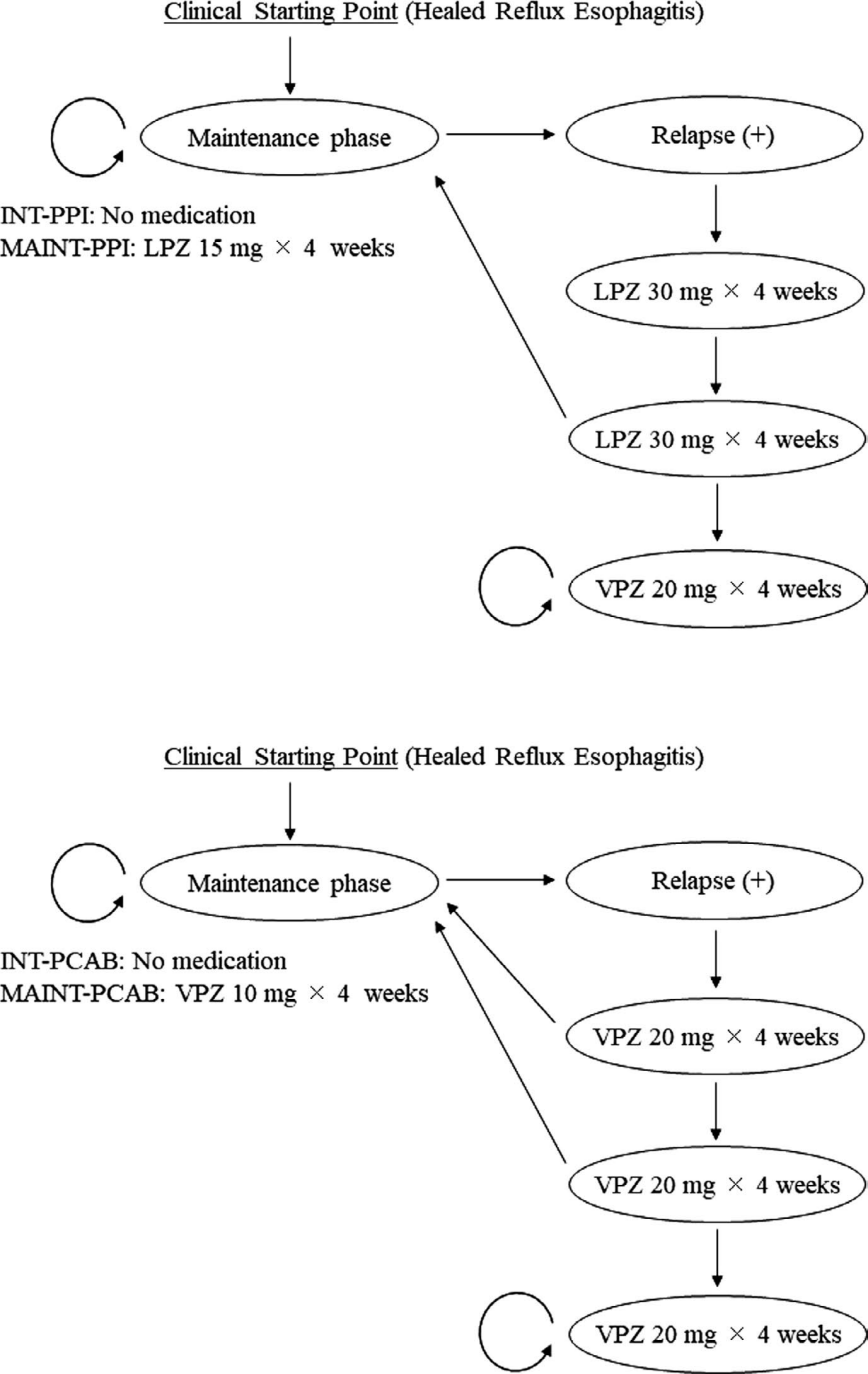
Schematic overview of the Markov model for alternative treatment strategies. Circles represent the monthly health states and the treatments that patients undergo. Arrows represent possible probabilistic transitions from one health state to another. Abbreviations: INT‐P‐CAB, intermittent potassium‐competitive acid blocker strategy; INT‐PPI, intermittent proton pump inhibitor strategy; LPZ, lansoprazole; MAINT‐P‐CAB, maintenance potassium‐competitive acid blocker strategy; MAINT‐PPI, maintenance proton pump inhibitor strategy; VPZ, vonoprazan

We employed Markov processes that followed patients on a monthly basis.^21^ Each health state (a 4 weeks period) was assigned clinical effects, such as the number of days without esophagitis (disease‐free days) and direct medical costs for the health services provided in each state. For every 4 weeks period, the probability of being in a particular state was multiplied by the associated clinical effects and costs. The resultant products for all states were summed and then added to the effects and costs of the previous 4 weeks. The chains were extended over a 12 months period to estimate the clinical effects and costs for 1 year after each initial management strategy. The Markov chains for alternative treatment strategies are shown in Figure 1. Table 1 shows the probability estimates in the analysis.

**TABLE 1:** Data used in model

Input variable	Value (Range)	References
Clinical events		
Healing rate (acute phase)		^17^
Lansoprazole (30 mg/d)		
4 wk	0.925 (0.879‐0.957)	
8 wk	0.955 (0.916‐0.979)	
Vonoprazan (20 mg/d)		
4 wk	0.966 (0.931‐0.986)	
8 wk	0.990 (0.965‐0.999)	
Relapse rate (maintenance phase)		
Intermittent (no therapy)	0.14/month (0.075‐0.24)	23, 24, 25, 26, 27
Lansoprazole (15 mg/d)	0.0302/month (0.0204‐0.0408)	^18^
Vonoprazan (10 mg/d)	0.00869/month (0.0034‐0.014)	^18^
Endoscopy to confirm relapse or healing	0 (0‐1)	

	Direct costs
Drugs	
Vonoprazan 20 mg	195.5 Yen
Vonoprazan 10 mg	130.3 Yen
Lansoprazole 30 mg	100.4 Yen
Lansoprazole 15 mg	57.6 Yen
Treatment	
Vonoprazan 20 mg × 4 wk^†^	6370 Yen
Vonoprazan 10 mg × 4 wk^†^	4410 Yen
Lansoprazole 30 mg × 4 wk^†^	3570 Yen
Lansoprazole 15 mg × 4 wk^†^	2450 Yen
Visit and physical examination	730 Yen
Routine blood tests	4030 Yen
Endoscopic examination	13 360 Yen

Note

One US dollar is equivalent to approximately 110 Japanese Yen.

^†^ Involves official charges for prescription and dispensing.

#### 2.2.1 Intermittent proton pump inhibitors strategy

Patients with healed esophagitis, who are in remission, receive no drug therapy unless symptoms recur (Figure 1). Current guidelines recommend a standard dose of PPI for eight weeks as an initial GERD treatment.^3^, ^7^ Therefore, patients with symptoms of recurrence are treated with LPZ (30 mg/d) for 8 weeks. After eight weeks of LPZ treatment, healed patients do not require further treatment or follow‐up visits. The patients with persistent disease after eight weeks of LPZ treatment are regarded as treatment failures. It is assumed that treatment failures are administered VPZ (20 mg/d) for the remaining study period, but that treatment continues to be unsuccessful. Patients who develop further recurrences are retreated in the same way as with the first recurrence.

#### 2.2.2 Intermittent potassium‐competitive acid blocker strategy

Patients with healed esophagitis, who are in remission, receive no drug therapy unless symptoms recur (Figure 1). Patients with symptoms of recurrence are administered acute VPZ treatment. The VPZ package insert stated that the usual treatment period should be up to 4 weeks, but may be extended for up to 8 weeks if the response to the initial treatment course was inadequate for the treatment of reflux esophagitis.^22^ This regimen was considered acceptable because a phase III trial reported that the healing of erosive esophagitis after four weeks of VPZ treatment (96.6%) was comparable to that after eight weeks of LPZ treatment (95.5%).^17^ Thus, patients who recurred are initially treated with VPZ (20 mg/d) for 4 weeks. After this treatment, the healed patients do not require further treatment or follow‐up visits, whereas the patients with persistent disease are treated with VPZ for another 4 weeks. After 8 weeks of VPZ treatment, the healed patients require no further treatment or follow‐up visits. The patients with persistent disease after 8 weeks of VPZ treatment are also regarded as treatment failures. The assumptions regarding treatment failure are the same as those in other strategies and are described above in the “Intermittent PPI strategy” section. Patients who develop further recurrences are retreated in the same way as with the first recurrence.

#### 2.2.3 Maintenance proton pump inhibitors strategy

Patients with healed esophagitis, who are in remission, receive maintenance treatment with LPZ (15 mg/d) (Figure 1). Patients with symptoms of recurrence during this maintenance regimen are administered acute LPZ treatment. This acute treatment entails administration of LPZ (30 mg/d) for 8 weeks. Afterward, the healed patients receive maintenance treatment with LPZ (15 mg/d), whereas the patients with persistent disease are regarded as treatment failures. The assumptions regarding treatment failure are the same as those in other strategies. Patients who develop further recurrences during the maintenance treatment with LPZ (15 mg/d) are retreated in the same way as with the first recurrence.

#### 2.2.4 Maintenance potassium‐competitive acid blocker strategy

Patients with healed esophagitis, who are in remission, receive maintenance treatment with VPZ (10 mg/d) (Figure 1). Patients with symptoms of recurrence during this maintenance regimen receive acute treatment with VPZ. This acute treatment entails initial administration of VPZ (20 mg/d) for 4 weeks. Afterward, the healed patients receive maintenance treatment with VPZ (10 mg/d), whereas the patients with persistent disease are treated with VPZ (20 mg/d) for another 4 weeks. Thereafter, the healed patients receive maintenance treatment with VPZ (10 mg/d), and the patients with persistent disease are considered treatment failures. The assumptions regarding treatment failure are the same as those in other strategies. Patients who develop further recurrences during the maintenance treatment with VPZ (10 mg/d) are retreated in the same way as with the first recurrence.

### 2.3 Probability values

The healing probabilities for acute treatment with VPZ and LPZ were derived from a randomized controlled trial comparing VPZ and LPZ for the treatment of endoscopically confirmed reflux esophagitis in Japan.^17^ For sensitivity analyses, 95% confidence interval (CI) data were also derived from this trial (Table 1).

The relapse rates for the intermittent strategies (no mediation) were derived from 6 to 12 month follow‐up studies.^23^, ^24^, ^25^, ^26^, ^27^ There was no significant difference in relapse rates of healed patients according to the type of acid‐suppressing drug.^23^ Thus, a relapse rate of 0.14 per month, which was the median of the reported probability rates, was used in the baseline analysis. Sensitivity analyses were performed within the range of the minimum to the maximum reported probability rates.

The relapse rates for the two maintenance strategies were derived from a randomized controlled trial comparing VPZ and LPZ maintenance treatments for healed erosive esophagitis in Japan.^18^ For sensitivity analyses, 95% CI data were also derived from this trial.

### 2.4 Costs

Primarily, a payer's perspective was chosen for analyzing costs; thus, the analysis included direct medical costs reimbursable by the Japanese National Health Insurance system (Table 1). The reimbursable services are summarized in Table 1. Official charges specified by the Japanese National Health Insurance system (as of April 2020) were used.

### 2.5 Willingness‐to‐pay data

The management of GERD should consider the patients' views or preferences for treatment (Table 2). There are several markers that can be used to represent the burden of illness from the patients' point of view. Willingness to pay or co‐pay is one such measure suitable for economic evaluation.^15^ Willingness‐to‐pay data in Japanese patients with GERD were derived from the published literature.^28^

**TABLE 2:** Willingness to co‐pay in Japanese patients with reflux esophagitis

Question	Cumulative percent (%)	References
The limit of reasonable medical bill (monthly co‐payment) only for treatment of reflux esophagitis (n = 700) (Yen)		
>5000	0	^28^
5000 or less	1	
4500 or less	2	
4000 or less	4	
3500 or less	5	
3000 or less	15	
2500 or less	20	
2000 or less	43	
1500 or less	54	
1000 or less	86	
500 or less	100	
The limit of an allowable additional medical bill (monthly co‐payment) to improve symptoms (only in patients who are not satisfied with the current treatment (n = 331) (Yen)		
>5000	8	^28^
5000 or less	17	
4500 or less	19	
4000 or less	23	
3500 or less	25	
3000 or less	34	
2500 or less	40	
2000 or less	54	
1500 or less	62	
1000 or less	88	
500 or less	100	

### 2.6 Calculations

The evaluated effects included the number of disease‐free days, the number of days for which medication was required, and the number of physician‐visiting days. Regarding the calculation of disease‐free days, the healing of esophagitis was assumed to occur according to an exponential function.^29^ Healing probabilities were based on the healing rates in clinical trials where healing was verified by endoscopy. However, in clinical practice, repeat endoscopies cannot be performed in most cases, and patients are usually managed based solely on symptom relief.^3^, ^7^ Therefore, it was assumed that the healing and recurrence of esophagitis were verified based on the symptomatic state assessed by the physician in the baseline analysis. Regarding the probability of endoscopy to confirm relapse or healing, sensitivity analyses were performed. It was assumed that there was one physician visit every four weeks during medical therapy. Direct medical costs were evaluated. Cost‐effectiveness ratios were calculated from the cost required to achieve clinical success (healing of esophagitis) per patient. Monthly self‐payments of company workers were also calculated. The study model was run for 12 cycles (4 week periods) to simulate one year follow‐up period. Discounting was not applied. Sensitivity analyses were performed to assess how the results varied according to the differing probability estimates within an acceptable range of values. All analyses were performed using the Microsoft Excel 2010 software program (Microsoft).

## 3 RESULTS

### 3.1 Effects

The expected effects are presented in Tables 3. Regarding the healing of esophagitis, the maintenance P‐CAB strategy was the most effective, followed by the maintenance PPI strategy; the intermittent PPI strategy was the least effective. The number of required medication days and the expected number of physician visits were fewest with the intermittent P‐CAB strategy.

**TABLE 3:** Expected effects and costs per patient over a 12 month period

	INT‐PPI	INT‐P‐CAB	MAINT‐PPI	MAINT‐P‐CAB
No. of days without esophagitis	279	305	320	334
No. of days with medication	67	37	336	336
No. of physician visits	2.4	1.3	12	12
Direct medical costs (Yen)	10 858	9380	39 046	61 878
Cost‐effectiveness ratio (Yen/d without esophagitis)	39	31	122	185
Monthly co‐payment† (Yen)	271	234	976	1547
Monthly additional co‐payment (Yen)			742 (above INT‐P‐CAB)	571 (above MAINT‐PPI)

Abbreviations: MAINT‐P‐CAB, maintenance potassium‐competitive acid blocker strategy; INT‐P‐CAB, intermittent potassium‐competitive acid blocker strategy; INT‐PPI, intermittent proton pump inhibitor strategy; MAINT‐PPI, maintenance proton pump inhibitor strategy.

^†^ The self‐pay ratio of medical expenses for company workers covered by health insurance is 30% of total direct costs

### 3.2 Cost‐effectiveness

Regarding the direct costs, the intermittent P‐CAB strategy was the least expensive, followed by the intermittent PPI strategy; the maintenance P‐CAB strategy was the most expensive. The cost‐effectiveness ratios showed that the intermittent P‐CAB strategy was the most cost‐effective, and the maintenance P‐CAB strategy was the least cost‐effective from a payer's perspective (Tables 3). The intermittent P‐CAB strategy was more effective and less costly than the intermittent PPI strategy, that is, the former was dominant in comparison.

### 3.3 Monthly medical bill (co‐payment) for patients

The monthly co‐payments for patients of the intermittent P‐CAB, maintenance PPI, and maintenance P‐CAB strategies were 234, 976, and 1547 Yen, which corresponded to payment amounts that 100%, 86%, and 43% of patients considered reasonable, respectively. Comparing maintenance PPI to intermittent P‐CAB strategies, and maintenance P‐CAB to maintenance PPI strategies, the additional monthly co‐payment for patients was 742 and 571 Yen, respectively, both of which corresponded to a payment that 88% of patients considered acceptable to improve symptoms (Tables 2 and 3).

### 3.4 Sensitivity analyses

**FIGURE 2:**
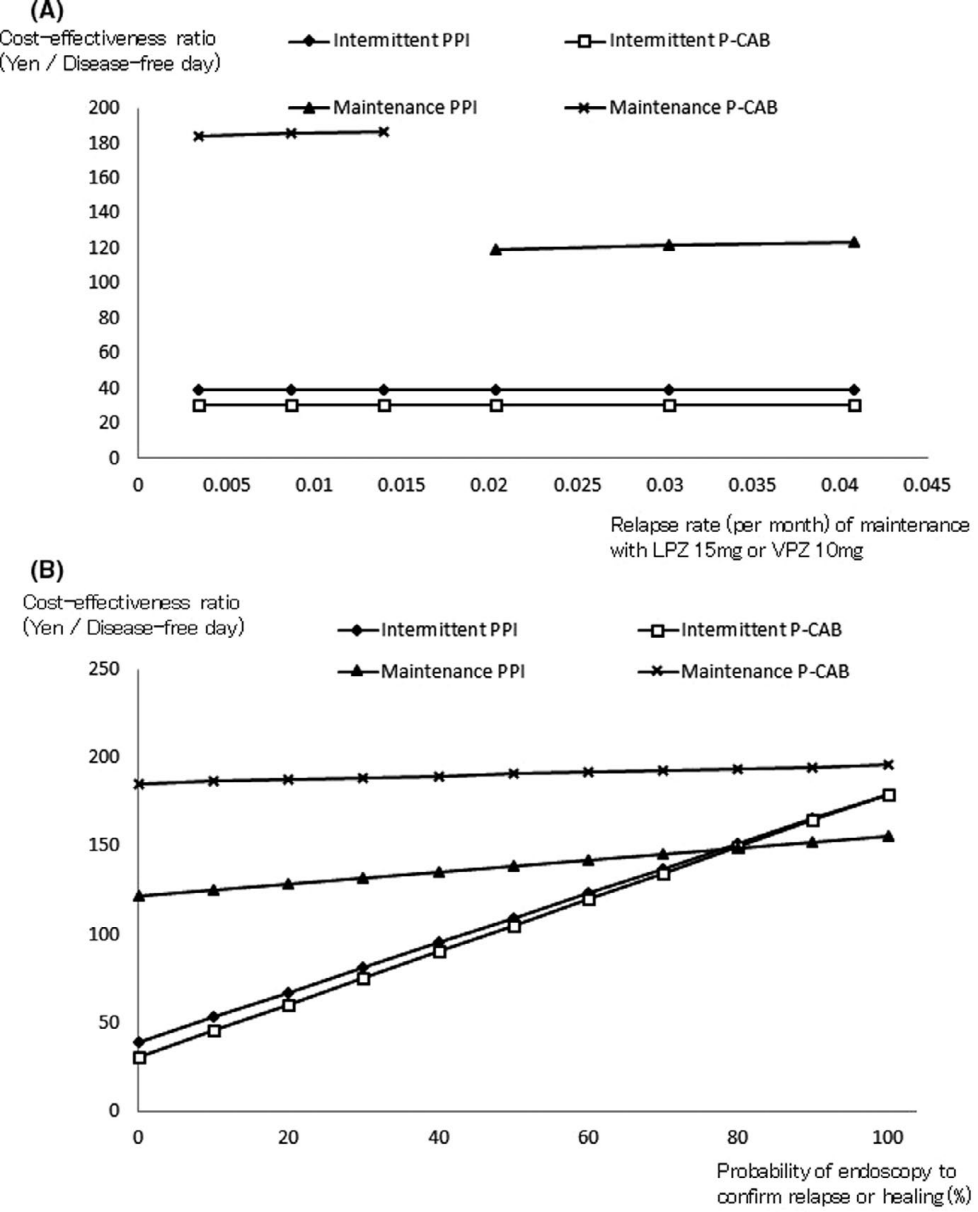
Sensitivity analyses on cost‐effectiveness testing the influence of (A) relapse rates during maintenance treatment with lansoprazole or vonoprazan, and (B) the probability of endoscopy to confirm relapse or healing. Abbreviations: LPZ, lansoprazole; P‐CAB, potassium‐competitive acid blocker; and PPI, proton pump inhibitor; VPZ, vonoprazan

One‐way sensitivity analyses regarding the healing and relapse rates across the entire range of estimates in Table 1 did not significantly alter the above‐described results. The cost‐effectiveness advantage of the intermittent P‐CAB strategy remained robust within the entire range of 95% CIs of the efficacy data (Figure 2). The cost‐effectiveness ratios of the intermittent strategies were rather sensitive to the probability of performing endoscopy to confirm relapse or healing. At a >80% probability of performing endoscopy to confirm relapse or healing, the maintenance PPI strategy was more cost‐effective than the intermittent P‐CAB strategy (Figure 2).

## 4 DISCUSSION

Proton pump inhibitors are now considered first‐line treatment for GERD, and PPI maintenance therapy is also recommended as an option for long‐term management.^3^, ^7^ However, recent studies have linked PPI use to various adverse effects, including bone fractures, chronic kidney disease, dementia, and ischemic strokes.^13^ Although GERD is well controlled by intermittent or on‐demand therapy in some patients,^10^, ^11^, ^12^ PPIs are often prescribed for extended periods without attempts to stop or reduce the dosage. In addition to the increased and, sometimes, inappropriate use of PPIs, growing concern regarding their safety has forced physicians to reconsider their utilization. This topic has been included in “Choosing Wisely,” a growing international campaign.^14^, ^30^, ^31^

VPZ is reported to achieve a more rapid and profound suppression of gastric acid secretion than PPIs.^16^ Clinical studies have demonstrated the remarkably high efficacy of VPZ.^17^, ^18^, ^19^

Gastroesophageal reflux disease is the most common gastrointestinal‐related diagnosis, and the cost of medical care for GERD is substantial.^1^ Thus, treatment strategies for GERD should be cost‐effective. This study was performed to investigate the cost‐effectiveness of alternative long‐term strategies for the management of erosive esophagitis in the era of a novel P‐CAB (VPZ) in Japan, in which four different strategies were compared: (a) intermittent PPI strategy; (b) intermittent P‐CAB strategy; (c) maintenance PPI strategy; and (d) maintenance P‐CAB strategy.

Regarding the healing of esophagitis, the maintenance P‐CAB strategy was the most effective, followed by the maintenance PPI strategy. The intermittent PPI strategy was the least effective. These results remained robust within the entire range of the 95% CIs of the efficacy data in the sensitivity analyses. The number of required medication days and the expected number of physician visits were fewest with the intermittent P‐CAB strategy. The expected number of days requiring medication per patient treated with the intermittent P‐CAB strategy was only 55% and 11% of those with the intermittent PPI strategy and with maintenance strategies, respectively. This information may be clinically important given the growing concerns regarding long‐term acid inhibition.^13^, ^14^, ^30^, ^31^

The intermittent P‐CAB strategy was the most cost‐effective from a payer's perspective. The intermittent P‐CAB strategy was more effective and less costly than the intermittent PPI strategy, that is, the former was dominant in comparison. In sensitivity analyses, these results remained robust within the entire range of 95% CIs for the efficacy data. One possible explanation is that the superiority of the intermittent P‐CAB strategy in terms of cost‐effectiveness is owing to the remarkably high efficacy of VPZ, which can heal most erosive esophagitis cases within a short time.^17^ Sensitivity analyses showed that the cost‐effectiveness of the intermittent strategies was rather sensitive to the probability of performing endoscopy. At a >80% probability of performing endoscopy, the maintenance PPI strategy was more cost‐effective than the intermittent P‐CAB strategy. However, previous reports suggest that repeat endoscopies in patients receiving acid‐inhibiting therapy did not essentially modify the management of GERD.^32^, ^33^ Under treatment with a potent acid inhibitor, symptom control is generally indicative of adequate mucosal healing. Thus, the majority of cases are usually managed based solely on symptom relief in clinical practice, which is the baseline analysis in this study.^3^, ^7^ In fact, a questionnaire survey on the management of GERD involving 435 physicians in Japan reported that only 29.5% of general practitioners consider endoscopy necessary in the management of GERD.^34^ Thus, the intermittent P‐CAB strategy appears to be the most cost‐effective strategy for the majority of patients in clinical practice.

The maintenance PPI strategy was more effective, but more costly than the intermittent P‐CAB strategy. The maintenance P‐CAB strategy was more effective, but more costly than the maintenance PPI strategy. In such situations, a relevant question is whether the extra cost is worth the extra effect. Theoretically, an incremental cost‐effectiveness ratio (additional costs divided by incremental effectiveness) can be calculated. For example, the incremental cost‐effectiveness ratio of the maintenance PPI strategy versus the intermittent P‐CAB strategy is calculated as (39 046‐9380)/(320‐305) = 1978 Yen/extra healthy day. However, patients and physicians have no yardstick to determine whether this value is meaningful. It is therefore reasonable that the analyses include the costs and consequences that may be borne by the patient.^15^ Several studies have employed cost‐utility analyses using quality‐adjusted life years as the outcome measure.^35^, ^36^, ^37^, ^38^ These studies have indicated that the utility value of a health state associated with GERD symptoms has a great impact on results, and is the key determinant for deciding cost‐effectiveness. The utility value is a quality‐of‐life measure with possible values between 0, representing death, and 1, representing perfect health. However, in those studies, utility value estimates often were not derived from patients directly, but were obtained from expert consensus or data from patients from other geographic areas with different disease severities. Moreover, the adopted utility values among those studies varied widely. For example, the utility value for the state with symptoms of GERD surprisingly varied from 0.97 to 0.56.^35^, ^36^, ^37^, ^38^ In this study, to incorporate the patients' views or preferences, the monthly medical bills (co‐payment) for patients covered by health insurance were evaluated. The monthly co‐payment for patients in the intermittent P‐CAB, maintenance PPI, and maintenance P‐CAB strategies corresponded to payment amounts that 100%, 86%, and 43% of patients considered reasonable, respectively. Additional monthly co‐payments for the maintenance PPI strategy above those for the intermittent P‐CAB strategy and for the maintenance P‐CAB strategy above those for the maintenance PPI strategy were considered acceptable to improve symptoms in 88% of patients. Therefore, from a patient‐centered economic endpoint, the intermittent P‐CAB strategy is superior to both maintenance strategies. However, if a patient is not satisfied with the symptom control afforded by the intermittent P‐CAB strategy, changing to a more effective maintenance strategy appears to be a reasonable option for the majority of patients. For maintenance treatments, the lowest degree of acid suppression required for symptom control should be the target criterion given growing concerns regarding long‐term acid inhibition.^13^, ^14^, ^30^, ^31^

A recent pharmacoeconomic analysis focused on the acute‐phase treatment of reflux esophagitis demonstrated that VPZ appears to be the drug of choice, and the model structure in this analysis formed the basis of that used in the present study.^20^ A cost‐utility analysis comparing VPZ versus esomeprazole or rabeprazole has been published.^38^ In this analysis, compared strategies were limited to continuous maintenance therapies, although the majority of erosive esophagitis is mild type, such as Los Angeles classification grade A or B. The present analysis is the first study to assess the cost‐effectiveness of alternative long‐term strategies for the management of reflux esophagitis, including continuous and discontinuous treatment with VPZ. It provides a basis for active patient participation in illness management, and the realization of patient‐centered medical practice by promoting conversations between physicians and patients.^14^, ^30^, ^31^ A limitation of this analysis is that the study model does not include generic medications. Generic drugs achieve blood concentrations similar to those of original drugs in healthy volunteers, but their comparative effectiveness has not been well evaluated. Studies comparing acid‐inhibitory effects of generic PPIs with the original PPIs including LPZ revealed that percentages of time pH >4 with some brands of generic PPIs were significantly lower than those with the original counterparts.^39^, ^40^ In addition, a comprehensive literature search did not identify any randomized controlled trials comparing the clinical efficacy of generic versus original PPIs, and reliable efficacy data of generic PPIs were not available. It was therefore decided not to include generic drugs in this study. Further analysis considering generic options would be needed to fully explore the cost‐effectiveness of real‐world treatment options.

## 5 CONCLUSIONS

The intermittent P‐CAB strategy was cost‐effective, and the number of days requiring medication was fewer than those with other strategies, which may be clinically beneficial. Therefore, the intermittent P‐CAB strategy appears to be the strategy of choice for the majority of reflux esophagitis patients in clinical practice. The maintenance PPI strategy was more effective, but more costly than the intermittent P‐CAB strategy. The maintenance P‐CAB strategy was more effective, but more costly than the maintenance PPI strategy. If a patient is not satisfied with the symptom control of the current strategy, he/she can switch to a more effective strategy, which appears to be a reasonable option for the majority of patients.

## CONFLICT OF INTEREST

The authors have stated explicitly that there are no conflicts of interest in connection with this article.
